# Effects of a Multidisciplinary Approach to Improve Volume of Diagnostic Material in CT-Guided Lung Biopsies

**DOI:** 10.1371/journal.pone.0140998

**Published:** 2015-10-19

**Authors:** Philip E. Ferguson, Catherine M. Sales, Dalton C. Hodges, Elizabeth W. Sales

**Affiliations:** 1 Department of Pathology, St. Bernards Medical Center, Jonesboro, AR, United States of America; 2 Doctors’ Anatomic Pathology Services, Jonesboro, AR, United States of America; 3 PathMD, Jonesboro, AR, United States of America; 4 Department of Public Health, Tulane University, New Orleans, LA, United States of America; 5 Department of Science and Mathematics, Arkansas State University, Jonesboro, AR, United States of America; University of Utah Health Sciences Center and ARUP Laboratories, UNITED STATES

## Abstract

**Background:**

Recent publications have emphasized the importance of a multidisciplinary strategy for maximum conservation and utilization of lung biopsy material for advanced testing, which may determine therapy. This paper quantifies the effect of a multidisciplinary strategy implemented to optimize and increase tissue volume in CT-guided transthoracic needle core lung biopsies. The strategy was three-pronged: (1) once there was confidence diagnostic tissue had been obtained and if safe for the patient, additional biopsy passes were performed to further increase volume of biopsy material, (2) biopsy material was placed in multiple cassettes for processing, and (3) all tissue ribbons were conserved when cutting blocks in the histology laboratory. This study quantifies the effects of strategies #1 and #2.

**Design:**

This retrospective analysis comparing CT-guided lung biopsies from 2007 and 2012 (before and after multidisciplinary approach implementation) was performed at a single institution. Patient medical records were reviewed and main variables analyzed include biopsy sample size, radiologist, number of blocks submitted, diagnosis, and complications. The biopsy sample size measured was considered to be directly proportional to tissue volume in the block.

**Results:**

Biopsy sample size increased 2.5 fold with the average total biopsy sample size increasing from 1.0 cm (0.9–1.1 cm) in 2007 to 2.5 cm (2.3–2.8 cm) in 2012 (P<0.0001). The improvement was statistically significant for each individual radiologist. During the same time, the rate of pneumothorax requiring chest tube placement decreased from 15% to 7% (P = 0.065). No other major complications were identified. The proportion of tumor within the biopsy material was similar at 28% (23%–33%) and 35% (30%–40%) for 2007 and 2012, respectively. The number of cases with at least two blocks available for testing increased from 10.7% to 96.4% (P<0.0001).

**Conclusions:**

The effect of this multidisciplinary strategy to CT-guided lung biopsies was effective in significantly increasing tissue volume and number of blocks available for advanced diagnostic testing.

## Introduction

### Background

Lung cancer is the most common cancer and leading cause of cancer death in the United States with an estimated 224,000 new cases and 159,000 deaths in 2014. [1–3] Historically, tumors have been divided into small cell carcinoma (15%) and non-small cell carcinoma/other sub-types (85%) for treatment purposes. [4] Recent advances in treatment of non-small cell carcinomas, including bevacizumab, premetrexed, erlotinib, and crizotinib, have brought hope for prolonged survival and quality of life in advance stage disease. [5]

These recent advances have also required pathologists to accurately sub-classify non-small cell carcinomas and conserve tissue for molecular/advanced testing. Non-small cell carcinomas can be sub-classified into multiple histologic sub-types, but are primarily classified as squamous cell carcinoma, adenocarcinoma, or large cell carcinoma. [6] This classification is typically done by morphology and immunohistochemistry (IHC) as needed.

Bevacizumab is contraindicated in the treatment of squamous cell carcinoma due to risk of life threatening pulmonary hemorrhage. Pemetrexed treated adenocarcinomas show improved outcomes. [5] EGFR mutated tumors comprise approximately 11% of lung adenocarcinomas and have a 70% response rate to erlotinib with prolonged survival. [7] Finally, ALK and ROS-1 mutated tumors (typically adenocarcinomas or large cell carcinomas) have high response rates (61–72%) and prolonged survival when treated with crizotinib. [8]

Only 30% of non-small cell carcinoma lung cancer patients are surgical candidates. Therefore, the diagnostic biopsy may be the only material available to diagnose and guide treatment for 7 out of 10 patients. [9] Optimal conservation and utilization of biopsy material is critical for sub-classification and identification of specific mutations, which may be responsive to targeted therapies. [10, 11]

Consensus guidelines and multi-organizational reports have stated the importance of a multidisciplinary strategy for optimal collection and utilization of diagnostic biopsy tissue, but few specific recommendations have been given to put into practice. [5, 12–14] The 2011 the International Association for the Study of Lung Cancer/American Thoracic Society/European Respiratory Society (ATS/ERS/IASLC) classification review was one of the first prominent publications to emphasize a multidisciplinary approach, but to our knowledge there is no documentation in the medical literature quantifying the effect of a multidisciplinary strategy to increase the volume of diagnostic tissue for molecular/advanced testing. The purpose of this study was to evaluate the effects of such a strategy developed and implemented at a community hospital (Saint Bernards Medical Center, Jonesboro, AR) in late 2008 for CT-guided biopsies.

### Multidisciplinary Strategy Development

As demands for advanced testing on small biopsy samples have significantly increased for EGFR mutations, and more recently ALK and ROS-1, the need for an effective strategy from a systemic standpoint was recognized, and a multidisciplinary team (pathology, interventional radiology, and oncology) was assembled to develop a practical strategy to optimally handle and allocate tissue for current and potential future testing.

Prior to 2008 interventional radiologists would perform a CT-guided biopsy and prepare touch preparations of the core tissue, which would be reviewed by a pathologist as part of a rapid onsite evaluation (ROSE) to determine adequacy. If tissue was inadequate, then additional biopsies (passes) would be performed by the radiologist until there was confidence diagnostic tissue had been obtained, at which time the procedure would be terminated. Biopsy material would then be placed in 10% formalin solution and typically submitted in a single cassette for histologic processing. IHC, fluorescence in-situ hybridization (FISH) or molecular diagnostic testing would be performed on tissue available in the remaining block or unstained slides when needed.

The purpose of the multidisciplinary strategy was to increase diagnostic tissue volume and maximize tissue available for ancillary testing. Input was obtained from interventional radiologists, oncologists, and pathologists to better understand each specialty’s needs and limitations, which resulted in a consensus three-pronged strategy for CT-guided biopsies. This strategy focused on the following:

- Once the radiologist is confident diagnostic tissue has been obtained, and if safe for the patient based on the radiologist’s clinical assessment, additional passes (biopsies) are performed to increase the volume of diagnostic tissue available for ancillary testing. A specific number of total biopsy passes or total tissue volume was not recommended, but at least two passes were implied. The consensus of the radiologists was to perform in the range of 2–4 biopsy passes depending on the location and characteristics of the lesion.
- Biopsy material is placed in multiple tissue cassettes (typically two) to minimize loss of diagnostic tissue if IHC or special stains are performed on one of the two blocks (leaving the second block available for ancillary testing).
- All tissue ribbons obtained during cutting at the microtome would be saved on unstained slides between levels, which may be used for IHC stains, FISH, or molecular studies.

The hypothesis for this study is that the change in approach to CT-guided biopsies increased material available for advanced testing. This study evaluates the effectiveness of the above described multidisciplinary team strategy for CT-guided lung biopsies and compares it to historic methodology with an emphasis on quantification of changes in diagnostic tissue volume. Additional effects including complications, tumor volume, need for ROSE, and rate of malignant diagnoses were also examined.

## Materials and Methods

This study is a retrospective analysis comparing CT-guided lung needle core biopsies performed in 2007 and 2012 to evaluate the sustained effect of the multidisciplinary strategy for obtaining and processing CT-guided lung biopsies, which was implemented in late 2008. Internal review board (IRB) approval was obtained through Saint Bernards Medical Center, Jonesboro, AR (Study #140801). Patient consent was waived, and patient data were anonymized and de-identified prior to analysis.

CT-guided biopsy reports were obtained for 209 consecutive biopsies in 2007, and 208 consecutive biopsies in 2012 at a single institution (Saint Bernards medical center) to represent steady-state practices before and after implementation of the multidisciplinary strategy. For the purpose of this study, only CT-guided lung biopsies were evaluated. The inclusion criteria were patients with a lung lesion who underwent a CT-guided biopsy resulting in a pathology report and had slides available for review. Exclusion criteria included cases from radiologists who did not perform CT-guided lung biopsies in both 2007 and 2012 at the study institution.

Patient medical records were reviewed and included imaging studies, demographics, procedural notes, and pathology reports. Lung biopsy microscopic slides were retrieved and analyzed for number of tissue fragments, minimum and maximum tissue fragment size, and total biopsy sample size by pathologist #1 (PEF). Total biopsy sample size was estimated to the nearest 0.1 cm using a transparent ruler containing tick marks to the nearest 0.05 cm (Ted Pella, Inc., Cat. No. 54480, Redding, CA). Pathologist #2 (EWS) estimated tumor percentage (area/volume estimate) visually on each slide in 5% increments.

It was assumed for the purpose of the study that the volume of tissue available for testing was directly proportional to specimen length on the microscope slide.

$$Volume=\pi (r^{2})\times Length$$

Technical aspects of obtaining CT-guided lung biopsies were unchanged between 2007 and 2012. Bard (C.R. Bard, Inc., Tempe, AZ) biopsy needles (19-gauge) were used exclusively for both 2007 and 2012 data sets maintaining a constant biopsy radius/diameter. Fluoroscopic CT-guidance was introduced in the radiology department 2009, which shortened the length of the procedure, but did not alter the technical methodology of obtaining biopsy material.

Complications for the purpose of this study were defined as secondary events caused by the biopsy procedure (e.g. pneumothorax or pulmonary hemorrhage), which resulted in additional treatment or prolonged observation (e.g. chest tube placement or admission for observation), and were identified through review of the interventional radiology biopsy procedure notes.

### Statistical Analysis

Statistical analysis was performed using GraphPad Prism Version 6 (GraphPad Software, Inc., La Jolla, CA). Ordinary one-way analysis of variance (ANOVA) was performed on both 2007 and 2012 data sets to determine if significant differences were present between radiologists. Unpaired t-test with Welch’s correction was used to evaluate changes in biopsy size between 2007 and 2012 for individual radiologists. The Mann-Whitney U test was used to evaluate the overall total biopsy length, and the Fisher exact test was used to evaluate statistical significance for the rate of malignant diagnoses, use of rapid onsite evaluation (ROSE), number of blocks submitted, and complication rate.

## Results

One-hundred and fifty CT-guided lung biopsies were performed in 2012 for mass lesions compared to 130 in 2007. Nine cases from 2007 were excluded from analysis. Four cases did not have microscopic slides available, and 5 cases were performed by radiologists who did not perform CT-guided lung biopsies at the study institution in 2012 for comparison. Thirty eight cases were excluded from the 2012 data analysis as they were performed by a radiologist who did not perform CT-guided lung biopsies at the study institution in 2007 for comparison. As a result, there were 121 cases from 2007 and 112 cases from 2012 being included in the study data set for analysis.

**Fig 1:**
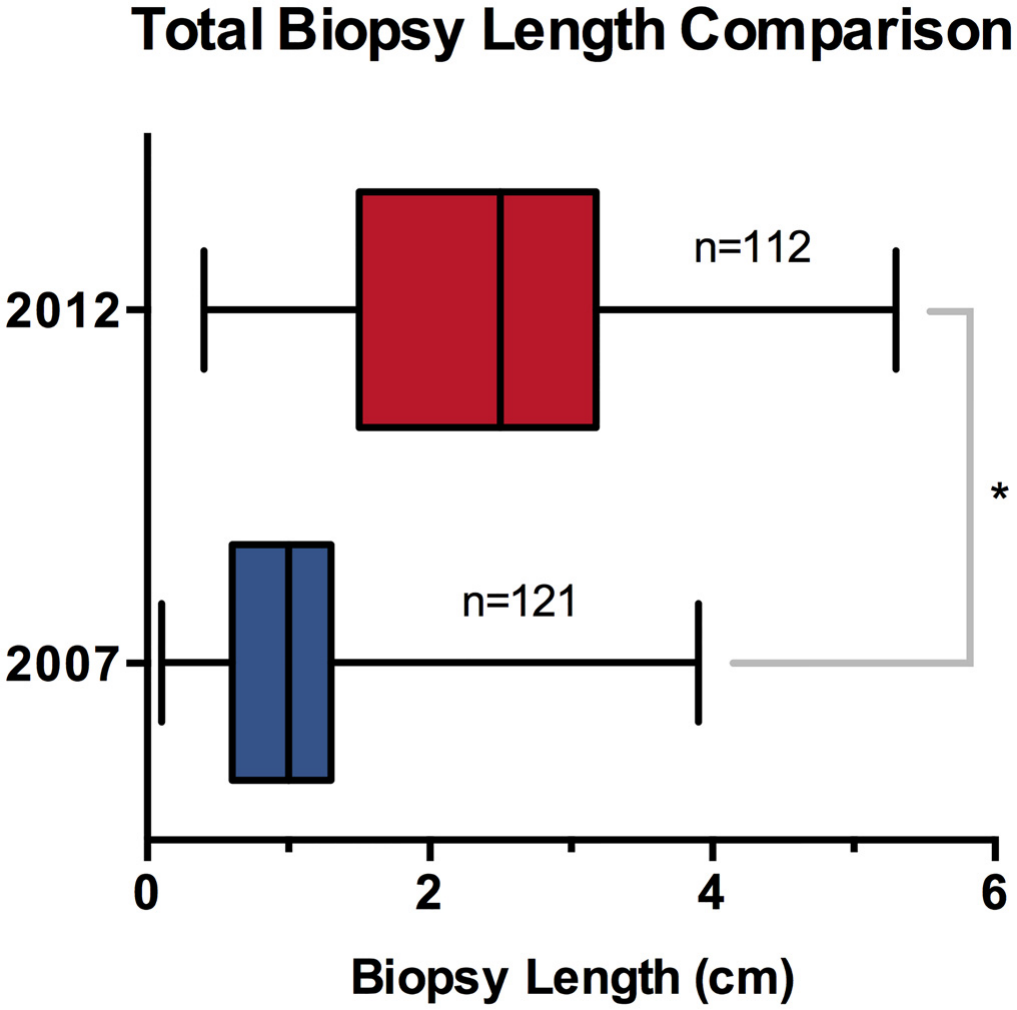
Box and whiskers (25%–75%) plot of total biopsy sample size comparing 2007 (blue) and 2012 (red). * P<0.0001 (Mann-Whitney U test).

**Fig 2:**
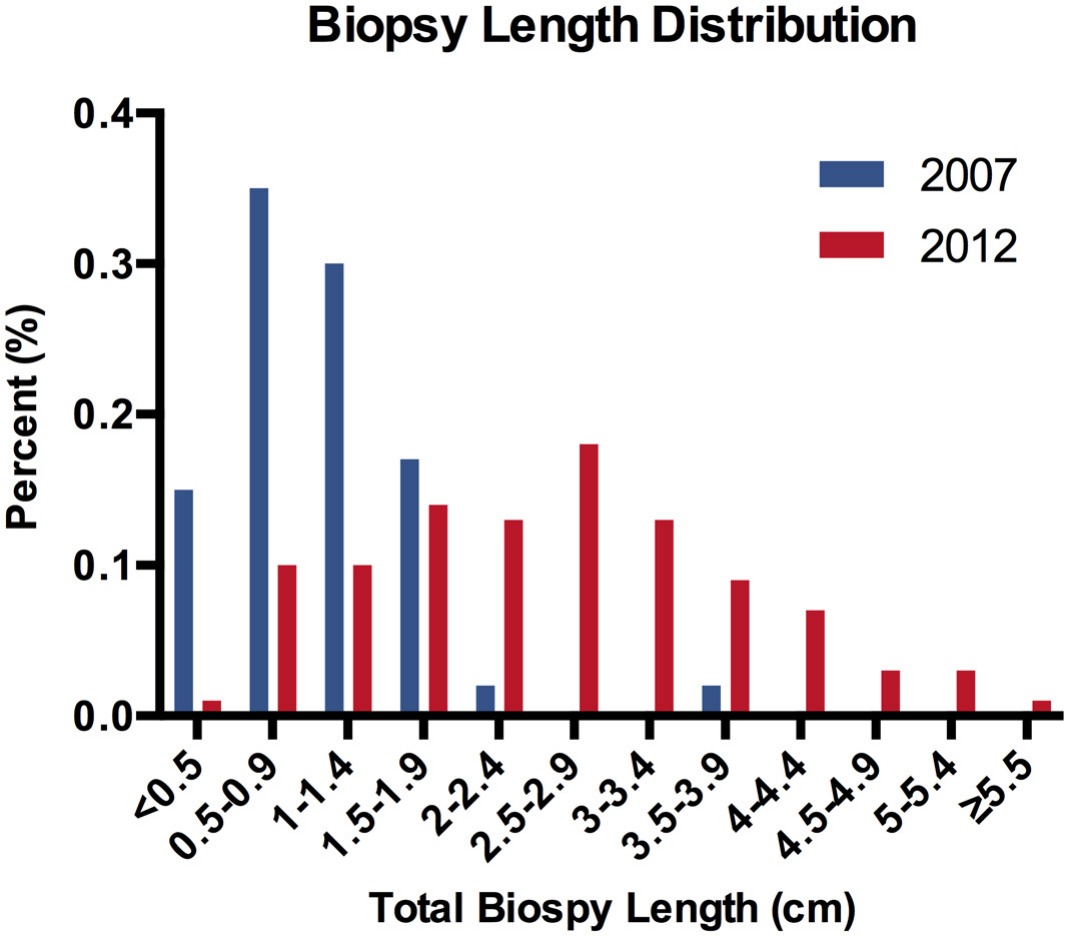
Distribution frequency of total biopsy sample size for 2007 (blue) and 2012 (red).

**Fig 3:**
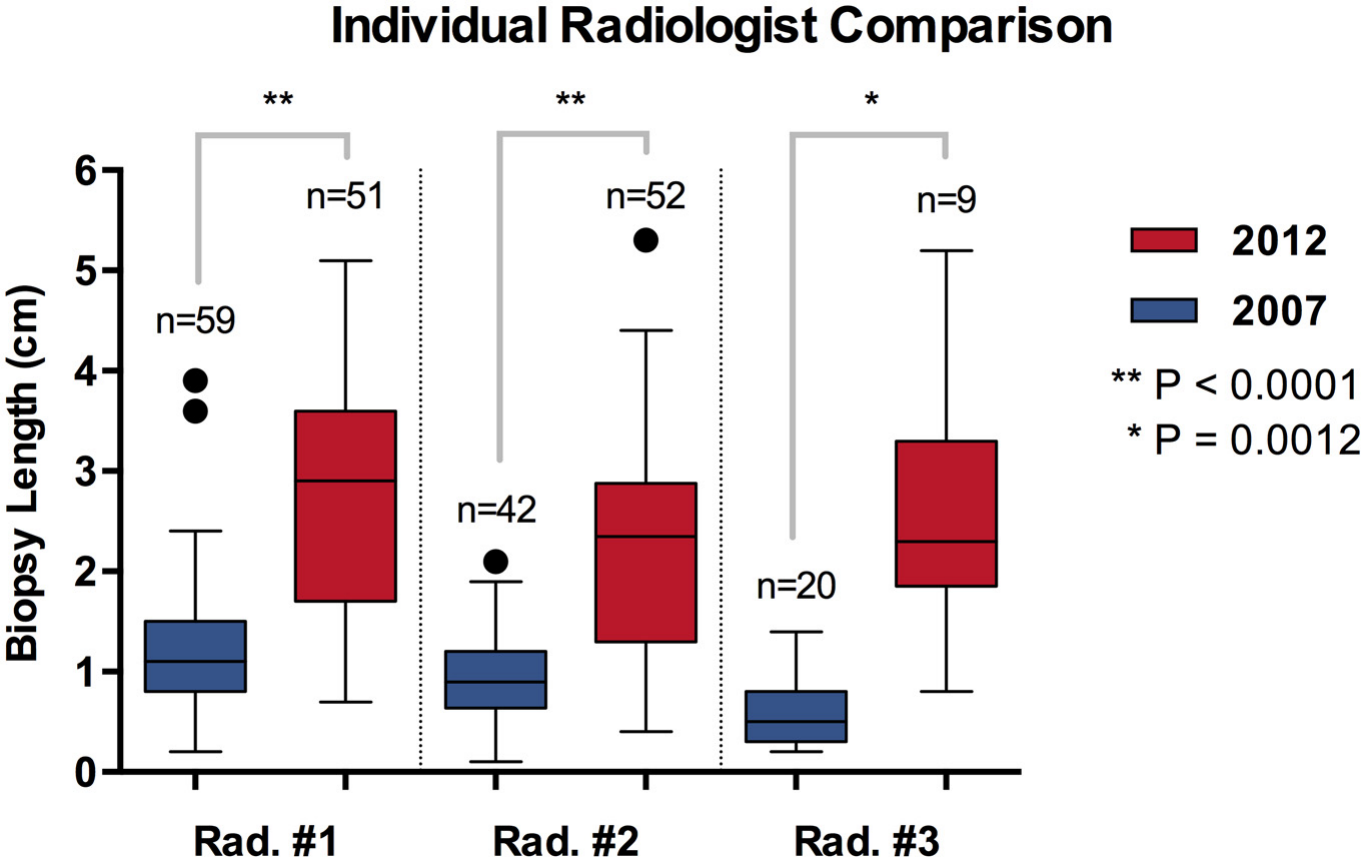
Box (25%–75%) and whiskers (minimum to maximum) plot for median biopsy length separated for individual radiologists between 2007 (blue) and 2012 (red).

The average total biopsy sample size increased from 1.0 cm (0.9–1.1 cm) in 2007 to 2.5 cm (2.3–2.8 cm) in 2012, which correlates to a 2.5 fold increase in average diagnostic tissue volume highlighted in Table 1 and Fig 1. Fig 2 shows the biopsy sample size distribution frequency as a function of biopsy sample size in 0.5 cm increments, and Fig 3 compares biopsy sample size for individual radiologists between 2007 and 2012.

**Table 1:** Study Results.

Variable	2007	2012
Total No. of CT-Guided Biopsies	130	150
No. of Cases Analyzed	121	112
Malignancy Rate (%)	62.0% (n = 75)	76.8% (n = 86)
Ave. Age (years)	66.1	66.9
- Male	65.0 (n = 66)	69.6 (n = 47)
- Female	67.4 (n = 55)	64.9 (n = 65)
Ave. Biopsy Length (95% CI)	1.0 cm (0.9–1.1 cm)	2.5 cm (2.3–2.8 cm)
Ave. Tumor Percentage (95% CI)	28% (23–33%)	35% (30–40%)
Number of Blocks Submitted		
- One	108 (89.3%)	4 (3.5%)
- Two	12 (9.9%)	108 (96.4%)
- Three	1 (0.8%)	0 (0%)
Complications (Clinically Significant)	18 (15%)	8 (7%)

Abbreviation: CI, Confidence Interval

Ordinary one-way ANOVA analysis of the 2012 data set showed no significant difference in biopsy sample size between all three radiologists (P = 0.1175), but the same analysis of the 2007 data set showed a significant difference among the radiologists (P<0.0001). This difference was identified to be between radiologist #3 and both radiologists #1 and #2 using Tukey’s multiple comparison test. The increase in biopsy size from 2007 to 2012 was statistically significant for each individual radiologist using an unpaired t-test with Welch’s correction. (Table 2)

**Table 2:** Individual Radiologist Performance.

		2007		2012	
Radiologist	N	Ave. Bx. Length	N	Ave. Bx. Length	P-value
Radiologist #1	59	1.2 cm (1.0–1.4 cm)	51	2.8 cm (2.4–3.1 cm)	< 0.0001
Radiologist #2	42	1.0 cm (0.8v1.1 cm)	52	2.3 cm (1.9–2.6 cm)	< 0.0001
Radiologist #3	20	0.6 cm (0.4–0.7 cm)	9	2.6 cm (1.6–3.6 cm)	0.0012
Combined	121	1.0 cm (0.9–1.1 cm)	112	2.5 cm (2.3–2.8 cm)	< 0.0001

Biopsy Length = combined total length of all biopsy fragments submitted. N = number of cases.

The percentage of cases diagnosed with malignancy increased from 62.0% (n = 75) in 2007 to 76.8% (n = 86) in 2012 (P = 0.0104). Average portion of biopsy material involved by tumor (volume estimation) was 28% (23–33%, 95% CI) and 35% (30–40%, 95% CI) for 2007 and 2012, respectively. (Table 1)

The spectrum of CT-guided lung biopsy diagnoses is shown in Table 3. The rate of non-small cell carcinoma, not otherwise specified (NOS) diagnoses decreased substantially from 29.3% to 4.6% between 2007 and 2012 (P<0.0001). Miscellaneous neoplasms included large cell carcinoma with neuroendocrine features (1), lymphoma (2), carcinoid (1), and pulmonary hamartoma (1). Non-diagnostic samples included necrosis without identifiable lesional tissue and benign lung, which was not representative of a radiologic abnormality.

**Table 3:** Biopsy Diagnosis Distribution.

Diagnosis	2007	2012
Total Cases Analyzed	121	112
Malignant	75	86
- Non-small cell carcinoma, NOS	22 (29.3%)	4 (4.6%)
- Adenocarcinoma	22 (29.3%)	38 (44.1%)
- Squamous Cell Carcinoma	22 (29.3%)	30 (34.8%)
- Small Cell Carcinoma	4 (5.3%)	4 (4.6%)
- Miscellaneous	2 (2.6%)	2 (2.3%)
- Metastasis	3 (4.0%)	8 (9.3%)
Suspicious for Malignancy	7	5
Benign	30	19
- Granulomatous Inflammation	18 (60%)	9 (47.4%)
- Inflammation / Fibrosis	12 (40%)	9 (47.4%)
- Miscellaneous	0 (0%)	1 (5.2%)
Non-Diagnostic / Inadequate	7	2

The only clinically significant complication identified from the biopsy procedure was pneumothorax requiring chest tube placement. In 2007, 15% (n = 18) of biopsies required chest tube drainage, but this decreased to 7% (n = 8) in 2012 (P = 0.065).

The number of biopsies submitted in at least two cassettes increased from 11% to 96% between 2007 and 2012 (P<0.0001).

## Discussion

### Biopsy and Tumor Volume

Average total biopsy sample size, which is directly proportional to volume, increased from 1.0 cm (0.9–1.1 cm) in 2007 to 2.5 cm (2.3–2.8 cm) in 2012 after implementation of the multidisciplinary strategy (Fig 1). The increased volume appears consistent for all radiologists (Fig 3). A total of 3 radiologists submitted lung biopsy material in both 2007 and 2012 with similar results. Radiologist #3 difference was statistically significant compared to both radiologists #1 and #2 for 2007 by one-way ordinary ANOVA analysis with Tukey’s multiple comparisons test. However, the same analysis of 2012 data did not show statistically differences between the 3 radiologists, implying better consistency among the interventional radiologists after implementation of the multidisciplinary biopsy strategy.

The proportion of biopsy material involved by tumor (Table 1) was similar at 28% (2007) and 35% (2012) while the biopsy volume increased 2.5 fold. Therefore, increasing the biopsy volume appears to have the desired and expected effect of increasing tumor volume available for testing. These results further emphasize the importance of maximizing the biopsy volume from the procedure to have maximum potential tumor volume available for advanced testing.

### Tissue Adequacy for Molecular Testing

Few studies have been published which evaluate the effect of tumor volume on the sensitivity of molecular studies for CT-guided lung biopsies. One study comparing molecular analysis on both CT-guided lung biopsy and the subsequent surgical resection specimen, demonstrated a failure rate of 11% in CT-guided biopsies to detect EGFR or KRAS mutations in a series of 18 patients.[15] In a recent study evaluating the effectiveness of crizotinib in ROS-1 mutated lung tumors, 4 of 26 (15%) FISH proven ROS-1 mutated tumors failed to have detectable mutations by next generation sequencing (NGS) when testing the remaining available biopsy material.[16] Large tertiary referral medical centers have documented an adequacy rate for molecular studies from CT-guided biopsies ranging from 67% to 91%.[17]

Unfortunately, characterization of selection bias for testing or remaining tumor volume available for testing was not described in these studies. Studies have typically documented procedural strategy in terms of “biopsy passes” and not in the form of biopsy or tumor volume. This issue raises concern that insufficient tumor may have been present for molecular testing. Future studies including biopsy and tumor quantification may be helpful to define goals for capturing adequate tissue during the pre-analytical tissue acquiring phase.

A recent article by Ferretti et al. demonstrated an increase in biopsy length of 15.6% from 10.9-mm to 12.6-mm after publication of the ATS/ERS/IASLC guidelines in 2011. Their adequacy for molecular testing of EGFR pyrosequencing increased from 85% to 98% over the same time period. Needle size used was an 18-gauge Angiotech BioPince (Medical Device Technologies Inc., Gainesville, FL), which has 49% more volume per unit length than a 19-gauge Bard biopsy needle used in the current study. [18] There is no description in their study of any institution-based educational or multidisciplinary strategy intervention during the time between the two data sets. The multidisciplinary-based strategy used in this study to obtain and share input from physician stakeholders in the patient’s care may be an important difference between the 15.6% increase in biopsy volume (Ferretti, et al.) compared to the 250% increase in this study.

No known studies using CT-guided lung biopsies have evaluated tumor volume and molecular testing sensitivity with correlation of testing in subsequent lung resection specimens. Such an analysis will be important, knowing the growing dependence of molecular tumor analysis in therapeutic decision making with 70% of lung cancer patients not being surgical candidates, and their entire treatment plan being dictated by information present in the biopsy material.

Advanced testing methodologies like next generation sequencing (NGS) are becoming more routine and show promise to be front line in the testing of lung carcinoma because the number of genes tested is very large at a relatively low cost.

This study takes the initial steps in a methodology strategy to increase tumor volume available for testing. An important future direction will be to verify the effect of strategies, such as this one, with regards to minimizing false negative results with advanced molecular testing in small biopsies.

### Rapid Onsite Evaluation

A secondary observation in this study was that the use of immediate cytologic assessment by a pathologist, commonly referred to as rapid onsite evaluation (ROSE), decreased significantly from 85% of cases in 2007 to 0% in 2012 among the radiologists included in this data set (P<0.0001). ROSE is commonly performed on cytology and needle core biopsy samples to determine if they contain diagnostic material from which to make a definitive pathologic diagnosis. If diagnostic tissue is not present, then additional tissue may be obtained immediately to increase the likelihood of a definitive diagnosis, and potentially preventing an unnecessary repeat biopsy.

After implementation of the multidisciplinary strategy in this study, interventional radiologists included in this analysis gained tacit knowledge that the overall diagnostic rate was at least equally sensitive after obtaining at least two needle core biopsies irrespective of whether a pathologist had performed ROSE or not. Subsequently, they changed from their historic practice and began to submit biopsies without ROSE. The perceived benefit by the radiologists was a shorter and possibly safer procedure. The diagnostic malignancy rate increased between 2007 and 2012, while the complication rate decreased (Table 1).

If a standard biopsy size or volume recommendation could be determined, which maximizes the likelihood of adequacy for diagnosis and advanced testing, then the use of ROSE could be significantly lowered and would result in significant cost savings. Additionally, a recent article by Rekhtman, et al. demonstrated that performing touch preparations on needle core biopsy tissue can decrease the total DNA content on average 15–50% depending upon how vigorous the touch preparations are made. [19]

These combined factors raise the question whether ROSE by a pathologist is necessary when an adequately sized biopsy is obtained, and if it may contribute to unnecessary expense, loss of valuable tissue for advanced testing, and increasing the procedure time that could contribute to complication risk. Additional study is needed in these areas.

### Number of Tissue Blocks Submitted

After implementation of the multidisciplinary strategy, the number of specimens with biopsy material placed in at least two blocks increased from 11% in 2007 to 96% in 2012 (P<0.0001). Tumor was present in both tissue blocks in 91% of the cases diagnosed with malignancy in 2012 (n = 86). Dividing biopsy tissue into two separate cassettes allows for less tissue to be consumed by immunohistochemistry than if all diagnostic tissue was submitted in a single cassette. Therefore, this strategy will on average increase the volume of tumor tissue remaining available for ancillary studies after the primary diagnosis has been made.

In theory, if one estimates half of the diagnostic biopsy tissue is consumed in making the primary diagnosis including IHC stains, and there is on average equal tumor present in both blocks, then submitting tissue in two cassettes would increase the amount of tumor available for molecular/advanced testing by 50% compared to submitting the biopsy sample in a single cassette. Combining this with the 2.5 fold increase in average tumor volume in this study would result in increasing tissue available for advanced testing 3.7 fold compared to the historic (pre-2008) strategy.

### Diagnosis

Table 3 highlights the distribution of diagnoses between 2007 and 2012. The most significant change over time was the decrease in the non-small cell carcinoma, NOS diagnosis rate from 29.3% to 4.6% (P<0.0001). This decrease is related to both increased utilization of immunohistochemistry by pathologists to assist in sub-classification, and in response to the clinician’s need to determine therapy given recent advances in chemotherapy (bevacizumab and pemetrexed), which are utilized in lung adenocarcinomas, and targeted therapies (crizotinib and erlotinib) for which mutations are most common in lung adenocarcinomas.

Table 1 also shows the rate of malignant (neoplastic) diagnoses increased from 62.0% in 2007 to 76.8% in 2012 (P = 0.0104). The increase in biopsy sample size may be the significant factor.

The incidence of small cell carcinoma (4.6–5.3%) is below the expected incidence of approximately 13%.[20] The reason for this apparent discrepancy is not clear from the data analyzed and is beyond the scope of this study, but could be related to choice of diagnostic methodology from the ordering physician. Small cell carcinomas tend to be more centrally located and may be more commonly biopsied through bronchoscopy or endobronchial ultrasound guided fine needle aspiration compared to CT-guided biopsy.

Granulomatous inflammation was a common diagnosis in this analysis. Histoplasmosis and blastomycosis are endemic to the geographical region in this study, which relates to the prominence of this finding.

The non-diagnostic / inadequate rate ranged between 5.8% in 2007 and 1.8% in 2012. This may be an underestimate of the false negative rate because some of the other benign diagnoses (e.g. inflammation/fibrosis) may represent un-sampled neoplasms. A significant portion of the decrease from 2007 to 2012 may be due to the increased neoplastic diagnosis rate from 2007 to 2012. This is a secondary observation of the data and not the primary endpoint of the study, and requires further study.

### Complications

Similar to previous interventional radiology studies, aside from chest tube placement for pneumothorax, complications are uncommon for CT-guided lung biopsies.[21] Some reports noted hemorrhage, but no cases of clinical significance were identified in our study. Pneumothorax not requiring chest tube placement was not considered clinically significant for the purpose of this study.

The rate of pneumothorax requiring chest tube placement decreased from 15% (n = 18) to 7% (n = 8) from 2007 to 2012 (P = 0.065). While this study was not designed to evaluate the underlying causes of this reduction, two main changes in workflow occurred during the intervening time between 2007 and 2012. First, fluoroscopic CT-guidance was installed in the CT-suite, which allows the radiologist to more quickly evaluate needle biopsy placement in near real-time at the bedside without having to walk between the patient and control room. Second, after changing the radiologist’s procedure to obtain more biopsy passes, the perceived tissue adequacy rate was high enough that radiologists did not routinely request ROSE by a pathologist. These two factors likely combined to reduce the length of time the biopsy needle is in the patient, which may be responsible for the decreased incidence of pneumothorax requiring chest tube placement.

Higher pneumothorax rates are associated with emphysema, smaller lesions, lesion depth, and operator experience.[22] Most studies did not correlate the length of time the biopsy needle is in the patient with risk of pneumothorax or chest tube placement. One study demonstrated that use of fluoroscopic CT-guided biopsies cut procedural time by 50% compared to conventional CT-guided biopsy.[23] Our data may indicate that the length of time the biopsy needle is placed in the patient could be another important risk factor of pneumothorax requiring chest tube placement. Unfortunately, this finding did not reach statistical significance for this study and lacks the data to further evaluate the possible association.

There is a wide range of reported incidences of pneumothorax for patients undergoing CT-guided lung biopsy, which has ranged from 9–54%.[24] In one study, up to 31% of patients undergoing CT-guided biopsy of small pulmonary nodules required chest tube placement.[21] The largest known study, which evaluated 15,865 cases from 4 different states for 2006, showed an overall average of 15% [CI, 0–50%] for any pneumothorax and 6.6% [CI, 0–25%] for pneumothorax requiring a chest tube. Our 2012 pneumothorax chest tube placement rate (7%) was consistent with large-scale published data.[25] Based on this data, there does not appear to be an increased risk of pneumothorax requiring chest tube placement after implementation of the multidisciplinary strategy, which should be reassuring to clinicians hesitant to perform additional biopsy passes out of fear of such complications.

## Conclusions

The findings of this study support the general consensus recommendations from multiple authors and organizations on the importance of having a multidisciplinary strategy for collection and handling of diagnostic lung biopsy tissue. Such an approach to CT-guided lung biopsies, which focuses on additional biopsy passes and submission of tissue in multiple cassettes, was effective in significantly increasing biopsy and tumor volume for potential molecular/advanced testing. Laboratories may want to consider strategies similar to those outlined in this study for small diagnostic biopsy material.

As molecular/advanced testing expands and becomes more commonplace, it will become important to develop best practice recommendations for obtaining and handling small biopsy specimens. Future studies correlating tissue adequacy for molecular testing with biopsy size and tumor composition may be helpful in defining best practices for tissue acquisition.

The multidisciplinary strategy implemented in this study does not appear to increase risk of complication, which in fact was actually decreased. There may also be additional benefits including higher rate of malignancy detection and less need for ROSE, which could have significant cost savings. Additional study of these specific areas is needed.
